# Effect of the At-CDC27a gene on *Nicotiana benthamiana* phenotype and accumulation of recombinant proteins

**DOI:** 10.3389/fpls.2022.1042446

**Published:** 2022-11-08

**Authors:** Lilya Kopertekh, Sven Reichardt

**Affiliations:** Institute for Biosafety in Plant Biotechnology, Julius Kühn-Institut (JKI) - Federal Research Centre for Cultivated Plants, Quedlinburg, Germany

**Keywords:** At-CDC27a, *Nicotiana benthamiana*, above-ground plant biomass accumulation, recombinant proteins, transient expression

## Abstract

In this study the anaphase promoting complex subunit CDC27a from *Arabidopsis thaliana* was introduced in the genome of *Nicotiana benthamiana* by *Agrobacterium tumefaciens*. The presence of the *At-CDC27a* gene facilitates plant biomass production. Compared to wild type *N. benthamiana* the leaf mass fraction of the best performing transgenic line At-CDC27a-29 was increased up to 154%. The positive effect of the At-CDC27a expression on leaf biomass accumulation was accompanied by an enlarged total leaf area. Furthermore, the ectopic expression of the At-CDC27a also affected cellular conditions for the production of foreign proteins delivered by the TRBO vector. In comparison to the non-transgenic control, the protein accumulation in the At-CDC27a-29 plant host increased up to 146% for GFP and up to 181% for scFv-TM43-E10. Collectively, the modified *N. benthamian*a plants developed in this study might be useful to improve the yield of recombinant proteins per biomass unit in closed facilities.

## Introduction

Transient expression holds the great potential as a platform for the large-scale production of recombinant proteins (Lomonossoff and D’Aoust, 2016). It provides a unique combination of features such as capability to produce complex proteins, relatively low capital costs, advanced technology for glycan modification, animal component- and endotoxin-free manufacturing and high scalability, with the production speed (Schillberg et al., 2019; Schillberg and Finnern, 2021). This method allows manufacturing of recombinant proteins in a matter of days making it very attractive for the production of emergency vaccines and diagnostics (Sainsbury, 2020). Transient expression is based on the inoculation of leaf tissue with *Agrobacterium tumefaciens* carrying the expression vector with the gene of interest (Kapila et al., 1997; Fischer et al., 1999). In the last decade the progress of this technology has been driven by the improving expression vectors and plant host (Kopertekh and Schiemann, 2019a). *N. benthamiana* is a preferred production host for transient expression (Goodin et al., 2008). This non-food, non-feed plant grows quickly in contained facilities and is susceptible to viral and bacterial infection (Bally et al., 2018). Different plant host cell engineering approaches such as reducing the impact of endogenous proteolysis (Mandal et al., 2016; Jutras et al., 2020), rebalancing leaf proteome (Robert et al., 2015), suppression of gene silencing (Matsuo and Matsumura, 2017; Matsuo and Atsumi, 2019; Matsuo, 2022) and modification of plant habitus (Fujiuchi et al., 2016) have proved to be effective to increase the recombinant protein accumulation. Current research efforts to alter the host plant architecture have been focused on modulation of cultural practice and manipulation of the *N. benthamiana* genome. In particular, several reports showed that optimisation of plant density and light quality can increase the amount of hemagglutinin (HA) in agroinfiltrated plants (Fujiuchi et al., 2017; Shang et al., 2018). Another study demonstrated positive effect of growth hormone 6-Benzylaminopurine (6-BAP) on H1 vaccine antigen yield (Goulet et al., 2019). An alternative approach to increase the efficiency of transient expression is based on the optimization of plant architecture. This can be done by stable integration of different regulatory genes taking part in photosynthesis, transcription, hormone metabolism, signalling and control of the cell cycle (Lima et al., 2017). Furthermore, cell cycle regulators were used to improve plant biomass production. Transgenic *N. benthamiana* plants expressing the At-CycD2 positive cell cycle regulator were shown to produce 143% more leaf biomass than non-transgenic plants. In comparison to wild-type the yield of recombinant proteins in the At-CycD2 plants was increased by 139% for the green fluorescent protein (GFP) and 157% for single chain variable fragment (scFv-TM43-E10) (Kopertekh and Reichardt, 2021).

The cell cycle consists of four phases, mitosis (M), postmitotic interphase (G1), DNA replication phase (S) and postsynthetic interphase (G2). A central role in controlling the cell cycle is played by the cyclin dependent kinases (CDKs). They are further controlled by various mechanisms, including degradation, binding to cyclins and inhibition (Inzé and De Veylder, 2006). One particularly ubiquitin ligase involved in the CDK degradation is the anaphase promoting complex (APC) (King et al., 1995; Peters, 2006). The APC is activated from early mitosis and remains functional during mitosis, G1 and early S phases (Heyman and De Veylder, 2012). This complex also has functions beyond the cell cycle. It has been reported that the APC is involved in different developmental processes including cellular differentiation, vascular development, shoot branching, root growth, hormone signalling, epigenetic regulation and embryogenesis (Saleme et al., 2021). In most eukaryotes, the APC is assembled from at least 13 different proteins (Barford, 2011). The subunit 3 of APC, also known as CDC27 subunit, is involved in protein-protein interactions and assembly of the structural module of the APC (Schreiber et al., 2011). In *Arabidopsis* two isoforms of the APC3 protein, CDC27a and CDC27b, have been identified (Pérez-Pérez et al., 2008). It has been already shown that overexpression of the At-CDC27a was beneficial for plant growth. For instance, *Nicotiana tabacum* plants carrying the *At-CDC27a* gene demonstrated an increase in stem and root biomass accumulation and leaf size when compared to wild type tobacco plants (Rojas et al., 2009). This accelerated growth has been attributed to an enhanced cell division and re-organization of the apical meristematic region. *In vitro* biochemical analysis using extracts from the At-CDC27a transgenic tobacco revealed a modified APC activity, which was manifested by an elevated ubiquitination of the mitotic cyclin (Rojas et al., 2009).

This study found that genetic modification of *N. benthamiana* with the cell cycle regulator gene *At-CDC27a* positively impacts recombinant protein production per unit of biomass.

## Materials and methods

### Plasmid constructs

The pLH-*35S-At-CDC27a* plant transformation vector (Kopertekh and Schiemann, 2019b) as well as the pJL-*TRBO-G* (TRBO-*GFP*) (Lindbo, 2007) and TRBO-*scFv-TM43-E10* (Kopertekh and Reichardt, 2021) constructs have been designed previously.

### Plant material

Transformation of *N. benthamiana* leaf explants with the pLH-*35S-At-CDC27a* binary vector were done according to the procedure described in Kopertekh and Reichardt (2021).

Transgenic T1 progeny seeds were harvested, surface-sterilised for 5 min with 70 % ethanol, washed 5 times with sterile water and sown on germination MS medium supplemented with 20 g/L sucrose, 0.5 g/L 2-(N-morpholino) ethanesulfonic acid (MES) (Roth, Karlsruhe, Germany), 0.8% (w/v) agar and 5 mg/L PPT (Duchefa, Haarlem, the Netherlands). Wild type *N. benthamiana* plants were germinated on the same medium without selection agent. PPT resistant seedlings containing T-DNA of the pLH-*35S-At-CDC27a* and control non-transgenic plants were transferred into the greenhouse and used in the subsequent experiments. Germinated seeds and plants were cultivated in the controlled environment chamber and greenhouse at 24°C with 16 h day/8 h night photoperiod.

### Molecular analysis of primary transformants

The transgenic status of primary regenerants was verified by PCR analysis. The genomic DNA was extracted essentially as described by Dale and Ow (1991) and amplified using primers specific to the *At-CDC27a*, *bar* and *GAPDH* genes (**Table S1**). The PCR reactions were carried out in the PTC-200 Peltier Thermal Cycler (Bio-Rad, Feldkirchen, Germany) at 94°C for 5 min followed by 30 cycles at 94°C for 1 min, 60°C for 1 min, 72°C for 1 min and 10 min of a final elongation at 72°C.

The copy number of T-DNA inserts in the At-CDC27a transgenic lines (At-CDC27a-6, At-CDC27a-22, At-CDC27a-29 and At-CDC27a-37) has been determined by Southern blot analysis. The protocol is provided in **Supplementary Materials**.

### Gene expression analysis

To determine the expression of the *At-CDC27a* gene total RNA was extracted from primary regenerants using the BioSELL RNA Mini Kit (Bio&SELL, Feucht, Germany). One µg of RNA was converted to cDNA by the random hexamer primer and Maxima Reverse Transcriptase following protocol suggested by supplier (Thermo Scientific, Waltham, USA). The RT-PCR has been carried out with the At-CDC27a-511-forw; At-CDC27a-511-rev primers specific to the *At-CDC27a* gene. The control endogenous glyceraldehyde 3-phosphate dehydrogenase (GAPDH) gene was amplified from cDNA by the GAPDH-238-forw, GAPDH-238-rev primers. The sequence of primers can be found in **Table S1**.

To estimate the expression level of GFP and scFv-TM43-E10 RNA from non-transgenic and *At-CDC27a-29* transgenic plants agroinfiltrated with the TRBO-*GFP* and TRBO-*scFv-TM43*-*E10* was isolated and used for cDNA synthesis following the above provided protocol. Quantification of transcripts was performed by qRT-PCR analysis as described earlier (Kopertekh and Schiemann, 2019b). Sequences of the gfp-forw/gfp-rev, TM43-E10-forw/TM43-E10-rev and ubi-forw/ubi-rev primers are listed in **Table S1**. Each sample was taken at 4 days after agroinfiltration (dpi) and was pooled from three middle agroinfiltrated leaves.

### Phenotypic analysis

The phenotypic analysis was performed on non-transgenic (wild type) and T1 progeny of the At-CDC27a transgenic plants. The transgenic T1 seeds were germinated on the MS medium supplemented with 5 mg/L PPT, whereas wild type seeds were germinated without selection pressure. Two weeks after sowing non-transgenic and PPT resistant transgenic seedlings were transferred to greenhouse and cultivated for four weeks. Four morphological parameters, plant height, number of leaves per plant, plant and stem biomass, were evaluated at this time point. The experiment included three replications. Each replication comprised of 10 transgenic and 10 control plants. The differences among the mean values were determined by unpaired T-test using SigmaStat software.

All leaves of the plant were collected at 4 weeks after planting and scanned. Leaf area of primary and secondary leaves was measured by ImageJ software and summarized to calculate the total leaf area for each leaf group of individual plant. The data were statistically analysed by the unpaired T-test using SigmaStat statistic software.

The cellular size-related characteristics such as epidermal cell area and number of cells per leaf have been calculated according to the protocol provided in Kopertekh and Reichardt (2021). Altogether the size of 587 and 683 epidermal cells for the At-CDC27a-29 and non-transgenic plants, respectively, was evaluated with ImageJ software.

### Production of recombinant proteins in the At-CDC27a transgenic and non-transgenic *N. benthamiana* plants

Agroinfiltration was performed following the procedure described by Kopertekh and Reichardt (2021). The samples for ELISA were collected from the middle leaves at 2, 4, 6 and 10 days after infiltration (dpi). Each sample was pooled from three leaves of one plant. The data were evaluated with Mann-Whitney test using SigmaStat statistic software. The description of the quantitative ELISA assay for GFP and scFv-TM43-E10 proteins can be found in Kopertekh and Reichardt (2021).

The detection of GFP (Kopertekh et al., 2004) and scFv-TM43-E10 (Kopertekh and Schiemann, 2019b) by Western blot has been described previously.

## Results

### Development and molecular analysis of *N. benthamiana* plants carrying the *At-CDC27a* gene

**Figure 1:**
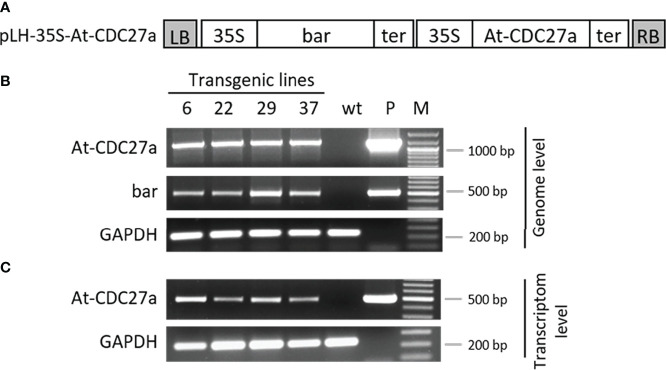
Molecular verification of the At-CDC27a transgenic lines. **(A)** Schematic diagram of the pLH-*35S-CDC27a* transformation vector. The pLH-*35S-CDC27a* binary vector contains the *bar* and *At-CDC27a* genes. Both genes are controlled by the CaMV 35S promoter and the appropriate terminator. The open boxes indicate the following sequences: bar, *bar* gene; At-CDC27a, *At-CDC27a* gene; 35S, 35S promoter; ter, terminator; LB, RB, left and right border of T-DNA, respectively. **(B)** PCR analysis of the At-CDC27a transgenic lines. Genomic DNA was isolated from the At-CDC27a-6, At-CDC27a-22, At-CDC27a-29 and At-CDC27a-37 transgenes and subjected to PCR analysis using the *At-CDC27a, bar* and *GAPDH* specific primers. Plasmid pLH-*35S-CDC27a* and genomic DNA from non-transgenic *N. benthamiana* served as positive and negative controls, respectively. **(C)** Expression analysis of the At-CDC27a transgenic lines. Total RNA extracted from the At-CDC27a-6, At-CDC27a-22, At-CDC27a-29 and At-CDC27a-37 transgenes were investigated by RT-PCR using specific primers for the *At-CDC27a* and *GAPDH*. Wild type *N. benthamiana* cDNA served as a negative control and the pLH-*35S-At-CDC27a* plasmid DNA served as a positive control. GeneRuler 100 bp Plus DNA marker (Thermo Scientific, Waltman, United States (M).

To understand whether the *At-CDC27a* gene affects the morphology of *N. benthamiana* plants, the pLH-*35S-At-CDC27a* vector (**Figure 1A**) containing the *bar* and the *At-CDC27a* genes was transformed in *N. bethamiana*. Agrobacterium-mediated transformation generated 52 independent regenerants, which were analyzed by PCR using oligonucleotides specific to the *bar* and *At-CDC27a* genes (**Table S1**). The predicted amplification products of 500 and 1200 bp for the *bar* and *At-CDC27a* genes, respectively, have been observed for 31 primary transformants confirming their transgenic nature. Representative DNA gels for lines At-CDC27a-6, At-CDC27a-22, At-CDC27a-29 and At-CDC27a-37 are shown in **Figure 1B**. **Figure S1** demonstrates the complete PCR analysis of 27 lines. Southern blot hybridization was used to determine the transgene copy number in the At-CDC27a-6, At-CDC27a-22, At-CDC27a-29 and At-CDC27a-37 transgenic events. This analysis revealed multiple T-DNA insertions in lines At-CDC27a-6 and At-CDC27a-37, whereas in lines At-CDC27a-22 and At-CDC27a-29 single T-DNA integration was observed (**Figure S2**).

The expression of the *At-CDC27a* gene in transgenic lines was evaluated by RT-PCR analysis using the At-CDC27a-511-forw and At-CDC27a-511-rev primers yielding a PCR product of 511 bp. The endogenous reference GAPDH gene was detected by the GAPDH-238-forw and GAPDH-238-rev oligonucleotides amplifying a 238 bp fragment. The sequence of the RT-PCR primers can be found in **Table S1**. The representative DNA gel shows the expected amplification products for the *At-CDC27a* and *GAPDH* genes in lines At-CDC27a-6, At-CDC27a-22, At-CDC27a-29 and At-CDC27a-37 (**Figure 1C**).

### Phenotypic analysis of the *N. benthamiana* transgenic lines carrying the *At-CDC27a* gene

**Figure 2:**
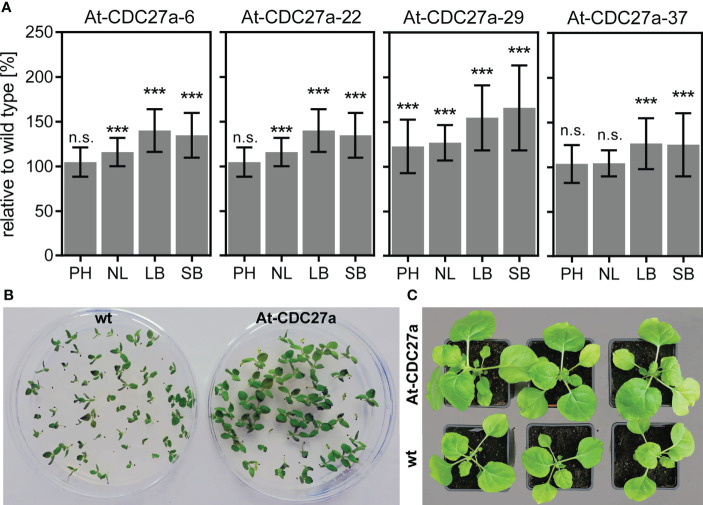
Phenotype of the At-CDC27a transgenic lines. **(A)** Assessment of plant height (PH), number of leaves (NL), leaf biomass (LB) and stem biomass (SB). The measurements were performed on the non-transgenic and T1 progeny of the At-CDC27a-6, At-CDC27a-22, At-CDC27a-29 and At-CDC27a-37 transgenic plants grown in the greenhouse for 4 weeks. The plant height, number of leaves and above ground biomass characteristics are expressed as a percent relative to the wild type. Values represent the means with standard deviation (n = 30). Asterisks indicate significance as determined by the unpaired T-test, with *** denoting p < 0.001. Not significant values are determined as ns. The At-CDC27a-29 and non-transgenic plants grown *in vitro* **(B)** and in soil **(C)**. The wild type *N. benthamiana* seeds and T1 progeny seeds of the At-CDC27a-29 line were germinated *in vitro* under nonselective and selective (5 mg/L PPT) conditions, respectively, and photographed at 12 days after sowing. The soil-grown plants were photographed at 21 days after planting.

T1 progeny of 14 At-CDC27a lines has been analyzed for their phenotype. Morphology-related abnormalities were not observed in the investigated transgenic lines. Four morphometric parameters such as plant height, number of leaves, leaf and stem biomass were evaluated. The T1 progeny of the At-CDC27a-6, At-CDC27a-22, At-CDC27a-29 and At-CDC27a-37 transgenic events exhibited an increased leaf and stem biomass accumulation compared to non-transgenic *N. benthamiana* (**Figure 2A**). Four weeks after planting, relative leaf biomass was significantly increased up to 140% for At-CDC27a-6, 138% for At-CDC27a-22, 154% for At-CDC27a-29 and 129% for At-CDC27a-37. Compared to wild type *N. benthamiana* stem biomass accumulation was 35, 32, 65 and 25% higher for lines At-CDC27a-6, At-CDC27a-22, At-CDC27a-29 and At-CDC27a-37, respectively. The height of the T1 progeny plants of the At-CDC27a-29 event was 122% of that for the non-transgenic plants. Additionally, the T1 progeny of the At-CDC27a-6, At-CDC27a-22 and At-CDC27a-29 transgenic events produced an increased number of leaves per plant (**Figure 2A**).

**Figure 3:**
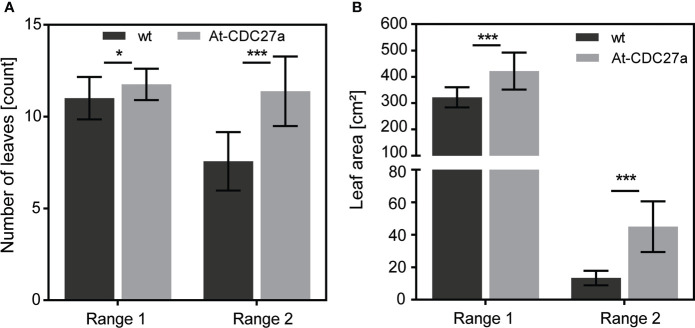
Leaf-related parameters of the At-CDC27a-29 transgenic line. Non-transgenic and transgenic plants were grown in the greenhouse conditions and number of leaves and leaf area per plant were determined after 4 weeks of cultivation. **(A)** Assessment of primary and axillary leaf number per plant. **(B)** Total leaf area for primary and secondary leaves. Values represent the means with standard deviation (n = 16). Asterisks indicate significance as determined by the unpaired T-test, with * and *** denoting p < 0.05 and p < 0.001, respectively.

The At-CDC27a-29 transgenic line was selected for further experiments due to its single locus T-DNA integration and increased above ground biomass accumulation. The T1 generation plants of this line showed an enhanced growth of young seedlings *in vitro* and during vegetative growth in greenhouse (**Figures 2B, C**). The At-CDC27a-29 and wild type *N. benthamiana* plants differed significantly in a number of primary and secondary stem leaves. Quantitative assessment of the primary leaf number four weeks after planting demonstrated that the At-CDC27a-29 genotype produced 11.8 ± 0.2 leaves compared to 11.0 ± 0.3 leaves produced by the non-transgenic control plants (**Figure 3A**). The same tendency was observed for the axillary leaves. On average, 11.4 ± 0.5 and 7.6 ± 0.4 leaves were quantified for the transgenic and wild type genotypes, respectively. The positive effect of the At-CDC27a expression on biomass production was accompanied by an increase in total leaf area per plant (**Figure 3B**). The total leaf area for primary stem leaves was significantly larger for the At-CDC27a-29 transgenic plant (421.7 ± 70.0 cm^2^) compared to non-transgenic control (321.8 ± 38.0 cm^2^). The total leaf area of axillary leaves for wild type plant (13.3 ± 4.4 cm^2^) was significantly smaller when compared to the At-CDC27a-29 (44.9 ± 15.6 cm^2^).

**Figure 4:**
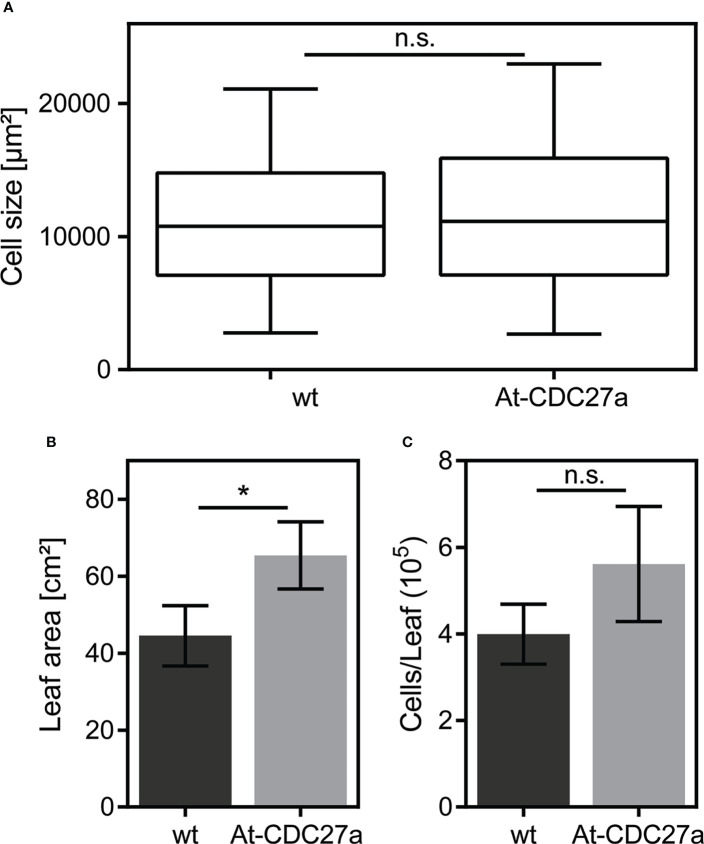
Cellular parameters of the At-CDC27a-29 and wild type *N. benthamiana* leaves. Epidermal cell **(A)**, leaf size **(B)** and number of cells per leaf **(C)** were determined in the third leaf of the At-CDC27a-29 and non-transgenic control plants (n=3). Values represent the means with standard deviation. Asterisks indicate significance with * p < 0.05. Not significant values are determined as ns.

Leaf growth is determined by cell proliferation and subsequent cell expansion under constant cultivation conditions. The total leaf area, the cell size and number of epidermal cells were analyzed in the third leaf of the At-CDC27a-29 and wild type *N. benthamiana* genotypes. This analysis revealed that the epidermal cell size of the At-CDC27a-29 leaves (11790 ± 265 µm^2^) was similar to that of the non-transgenic control (11114 ± 217 µm^2^) (**Figure 4A**) and the total leaf area of third leaf was increased in the At-CDC27a-29 plants compared to wild type (**Figure 4B**). Based on these two parameters the mean epidermal cell number per leaf was calculated as the ratio of leaf area to mean leaf epidermal cell area. The average mean for epidermal cell number per leaf in the At-CDC27a-29 line (5.62x10^5^ ± 0.77) was higher as that of the non-transgenic control (3.99x10^5^ ± 0.40). Although this difference was not statistically significant (**Figure 4C**).

### Expression of recombinant proteins in the At-CDC27a-29 line

**Figure 5:**
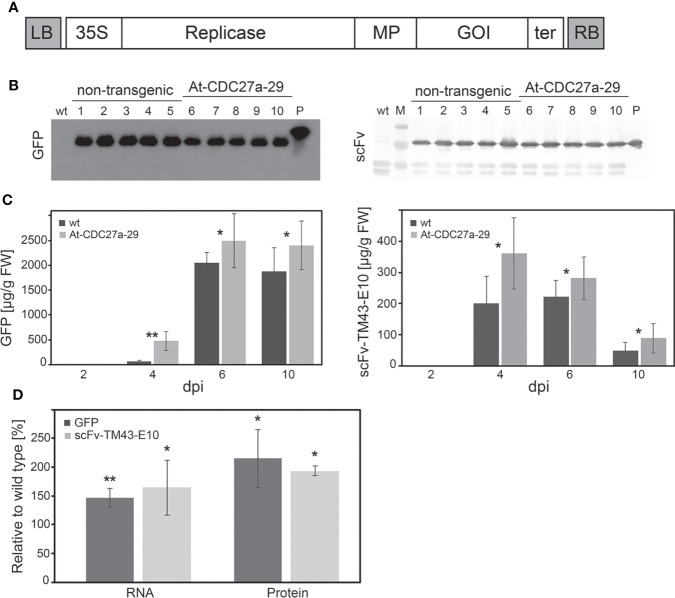
Transient production of recombinant proteins. **(A)** Schematic diagram of the TRBO expression vector. Open boxes represent the following sequences: 35S, CaMV 35S promoter; Replicase, RNA-dependent RNA polymerase; MP, 30 kDa protein involved in virus movement; GOI, gene of interest (GFP, scFv-TM43-E10); ter, 3’ terminator sequence; LB, RB, left and right border of T-DNA, respectively. **(B)** Western blot analysis of GFP and scFv-TM43-E10 accumulation. The leaf samples were collected from the At-CDC27a-29 and non-transgenic *N. benthamiana* plants agroinfiltrated with the TRBO-*GFP* and TRBO-*scFv-TM43-E10* expression constructs at 6 dpi. Six and one µg of total proteins for scFv-TM43-E10 and GFP plant extracts, respectively, were separated on 12% SDS-PAGE and analysed for the presence of scFv-TM43-E10 and GFP proteins by immunoblot. Lanes: 1-5, total protein extracts from the agroinfiltrated non-transgenic leaf material; 6-10, total protein extracts from the agroinfiltrated At-CDC27a-29 leaf material. P: GFP with His Tag (Biozol, Eching, Germany) and scFv-TM43-E10 (protein preparation extracted from *E coli* ) control proteins. Wt: total protein extract from the non-agroinfiltrated wild type *N. benthamiana* plant. M: PageRuler Prestained Protein Ladder (Thermo Scientific, Waltham, USA). **(C)** Time course analysis of GFP and scFv-TM43-E10 production. Leaf samples of the At-CDC27a-29 and non-transgenic plants agroinfiltrated with the TRBO-*GFP* and TRBO-*scFv-TM43-E10* expression vectors were collected on 2, 4, 6 and 10 dpi and subjected to ELISA to estimate protein accumulation. Each sample is a pooled sample generated by combining three infiltrated spots from middle leaves of one plant. For each time point 6 and 10 samples for GFP and scFv-TM43-E10, respectively, are shown. Values represent the means with standard deviation. **(D)** Quantification of RNA and protein levels of GFP and scFv-TM43-E10. Total RNA and soluble proteins from the At-CDC27a-29 and wild type agroinfiltrated leaves were extracted at 4 dpi and investigated by qRT-PCR and ELISA to estimate RNA and protein accumulation, respectively. For RNA quantification 10 samples were evaluated for each variant (wt and At-CDC27a-29 plants). For protein quantification 6 and 8 samples for GFP and scFv-TM43-E10, respectively, were analysed. Each sample is a pooled sample generated by combining three infiltrated spots from middle leaves of one plant. The values are expressed as a percent relative to the wild type and represent the means with standard deviation. Asterisks indicate significance as determined by the Mann-Whitney test, with *and ** denoting p < 0.05 and p < 0.01, respectively.

To evaluate the suitability of the At-CDC27a-29 line for recombinant protein production the non-transgenic and At-CDC27a transgenic plants were infiltrated with *A. tumefaciens* carrying the TRBO-*GFP* and TRBO-*scFv-TM43-E10* constructs (**Figure 5A**). Six days after inoculation the total protein fractions were extracted and separated on a SDS-PAGE under reducing conditions. Western blot analysis confirmed the integrity of the recombinant proteins. In the transgenic plants as well as in the control plants the produced proteins had the expected size of 27 kDa for GFP and 29 kDa for scFv-TM43-E10 (**Figure 5B**).

To analyze the expression of recombinant proteins over time, the leaf tissue of the transgenic and the control plants was harvested at 2, 4, 6 and 10 days after infiltration and the level of recombinant proteins was determined by ELISA. The expression of the recombinant proteins (GFP and scFv-TM43-E10) revealed an enhanced expression in plants carrying the At-CDC27a-29 gene for all time points. The accumulation of scFv-TM43-E10 peaked at 4 dpi with 200 ± 87 and 361 ± 90 µg/g fresh weight in wild type and transgenic plants, respectively. The highest GFP production was observed at 6 dpi approaching 1708 ± 210 µg/g fresh weight in non-transgenic and 2491 ± 542 µg/g fresh weight in modified *N. benthamiana* (**Figure 5C**).

To investigate the expression of *gfp* and *scFv-TM43-E10* transcript levels, total RNA was extracted from the agroinfiltrated leaf tissue at 4 dpi and analysed by qRT-PCR. The relative *gfp* mRNA level detected in leaf samples of inoculated At-CDC27a-29 plants was increased up to 147% compared with the wild type *N. benthamiana* plants (**Figure 5D**, **Table S2**). The relative quantity of the *scFv-TM43-E10* RNA was increased up to 165% in comparison to non-transgenic control. The increased *gfp* and *scFv-TM43-E10* RNA accumulation in the transgenic plants correlated with the detected recombinant proteins levels. Compared to the control plants, recombinant protein production in the At-CDC27a-29 plants was increased up to 215% for GFP and up to 197% for scFv-TM43-E10 (**Figure 5D**, **Table S2**).

## Discussion

In this study gene encoding the CDC27a subunit of the APC complex was introduced into the genome of *N. benthamiana* by *A. tumefaciens*-mediated transformation. Thirty-one independent regenerants were obtained in the presence of the selective agent and confirmed by specific PCR. The phenotypic characterisation of the At-CDC27a genotype and the production of recombinant proteins in the selected At-CDC27a-29 transgenic line revealed two characteristics that could be useful for molecular farming application. The first characteristic is an enhanced above-ground biomass production in T1 progeny of the At-CDC27a-6, At-CDC27a-22, At-CDC27a-29 and At-CDC27a-37 transgenic events. This result is consistent with the previously published data for a number of genes involved in the cell cycle control. For example, in *N. tabacum* plants the *At-CycD2* gene caused accelerated development and increased above-ground biomass accumulation (Cockcroft et al., 2000). Similar results were shown for the transgenic *A. thaliana* plants harbouring a putative *CycD2* gene from *Triticum aestivum* (*Triae-CycD2)*. Compared to the wild type control they developed more leaves and flowers (Wang et al., 2006). The introduction of the APC subunits from Arabidopsis, (At-CDC10 and At-CDC27a) into *N. tabacum* genome resulted in a significant increase of stem and leaf biomass and early induction of flowering (Rojas et al., 2009; Lima et al., 2017). Data from several laboratories suggest that the beneficial effect of the *At-CycD2*, *Triae-CycD2* and *At-CDC27a* genes on leaf biomass accumulation is based on an acceleration of cell division and the reorganization of the shoot apical meristem (Boucheron et al., 2005; Wang et al., 2006; Rojas et al., 2009). In the case of the At-CDC27a, several mechanisms have been suggested to explain the impact of the CDC27 gene on the plant phenotype. First, overexpression of the At-CDC27a subunit may led to a mild enhancement of APC-dependent cyclin degradation, followed by an accelerated cell cycle transition and subsequent cell division in the meristem. Alternatively, overexpression of the At-CDC27a could inhibit the activated APC/Cdh1 complex leading to an increased cyclin G1 accumulation and faster G1/S transition (Rojas et al., 2009).

Several papers reported that under constant growth conditions the leaf area at a given rank on the plant was more associated to its final epidermal cell number than to its final epidermal area (Granier et al., 2000; Cookson et al., 2005). Supporting these observations, the increase in leaf area and epidermal cell number were observed in tobacco plants ectopically expressing the *At-CDC27a* gene (Rojas et al., 2009). In our study the At-CDC27a *N. benthamiana* plants displayed an increased leaf area. However, the mechanism of this phenomenon remains to be investigated.

The second feature of the At-CDC27a genotype, which is relevant to molecular farming application, is the intercellular environment supporting the TMV-mediated recombinant protein expression. In the presence of the *At-CDC27a* gene the enhanced accumulation of GFP and scFv-TM43-E10 antibody fragment has been detected in leaves agroinfiltrated with the appropriate constructs. In recent years several publications have reported the positive impact of cell cycle regulators from plants and viruses on transient protein expression in *N. benthamiana*. In particular, co-expression of virus cell cycle regulator genes such as *Clink* gene from Banana bunchy top virus, *REn* gene from Tomato leaf curl virus, *RepA* genes from Tobacco yellow dwarf virus and Maize streak virus, elevated the GUS accumulation about 2 or 3-fold (Norkunas et al., 2018). Similar increases in GFP and scFv-TM43-E10 yield were observed in *N. benthamiana* plants co-infiltrated with the plant-derived cell cycle regulators At-CycD2 and At-CDC27a and full TMV-based vector carrying GFP and scFv-TM43-E10 (Kopertekh and Schiemann, 2019b). Another study confirmed the positive effect of stably integrated *At-CycD2* on production of GFP and scFv-TM43-E10 provided by the deconstructed TMV-based TRBO vector (Kopertekh and Reichardt, 2021).

The interaction of TMV with the host cell cycle has not been well studied. The results currently presented in the literature are quite fragmentary. Previous studies with *Nicotiana sylvestris* protoplasts have shown that the initial binding of the TMV virions to plant protoplasts is determined by the cell cycle stage of the host cell (Gould et al., 1981). Subsequently, microarray analysis of the TMV-infected tobacco plants revealed 5 up-regulated transcripts associated with the cell cycle control points. Most importantly, there was a 138-fold increase in RNA accumulation of the cell division checkpoint control protein RAD9A, suggesting its involvement in the TMV infection process (Jada et al., 2014). Another work revealed that the interaction of the Arabidopsis cell-division-cycle protein 48 (CDC48) with the TMV movement protein can modulate virus replication and cell-to-cell movement (Niehl et al., 2012; Niehl et al., 2013). In *N. benthamiana* leaves co-infiltrated with the plant-derived cell cycle regulators At-CycD2, At-CDC27a and TMV an increased accumulation of TMV RNA was observed suggesting that viral replication or intercellular movement may be altered (Kopertekh and Schiemann, 2019b). To summarize, there is a growing body of evidence showing the positive effect of viral and plant cell cycle regulator genes on transient expression in *N. benthamiana*. The cell cycle proteins derived from the ssDNA plant viruses could enhance accumulation of GUS provided by the non-viral pEAQ expression vector (Norkunas et al., 2018). In respect to the plant-derived proteins controlling cell cycle only combination with the TMV-based vectors has been analysed (Kopertekh and Schiemann, 2019b). The feasibility of this strategy for plant cell cycle regulators combined with the non-viral vectors still has to be investigated.

Two parameters of agroinfiltration technology, the biomass yield and amount of recombinant protein per square meter, are critical for the efficiency of the process (Walwyn et al., 2015). Under our experimental conditions, 80 plants per square meter, 0.75 kg leaf biomass for non-transgenic and 1.04 kg for At-CDC27a-29 genotypes, can be obtained during one growing cycle (**Table S3**). At GFP accumulation levels of 1.28 g/m^2^ in the unmodified host and 2.59 g/m^2^ in the modified host the greenhouse productivity can be increased by a factor of 2. For the scFv-TM43-E10 protein 150.4 mg/m^2^ and 375,2 mg/m^2^ were calculated for the wild type and At-CDC27a-29 genotypes, respectively. Therefore, the greenhouse productivity for the scFv-TM43-E10 can be increased by a factor of 2.5.

Thus, the present study showed that the ectopic expression of the *At-CDC27a* gene in *N. benthamiana* altered the biomass accumulation and cellular environment in transgenic plants. These characteristics may have practical potential to increase recombinant protein yield in contained facilities.

## Data availability statement

The original contributions presented in the study are included in the article/**Supplementary Material**. Further inquiries can be directed to the corresponding author.

## Author contributions

LK contributed to the study conception, analysis and interpretation of the results. SR performed statistical analysis of data and designed figures. All authors contributed to the article and approved the submitted version.

## Funding

This research was financed in frame of the self-funded JKI project by the German Federal Ministry of Food and Agriculture (BMEL).

## Acknowledgments

We thank Dr. Ferreira (Universidade Federal do Rio de Janeiro, Brazil) and Dr. J. Lindbo for providing the pGSV435sAtCDC27a and pJL-TRBO-G (Addgene plasmid # 80083; http://n2t.net/addgene:80083; RRID: Addgene_80083) plasmids, respectively. The technical support of Cornelia Freyer, Nadja Engelhardt and Bärbel Apel is also greatly acknowledged.

## Conflict of interest

The authors declare that the research was conducted in the absence of any commercial or financial relationships that could be construed as a potential conflict of interest.

## Publisher’s note

All claims expressed in this article are solely those of the authors and do not necessarily represent those of their affiliated organizations, or those of the publisher, the editors and the reviewers. Any product that may be evaluated in this article, or claim that may be made by its manufacturer, is not guaranteed or endorsed by the publisher.
