# Supplementary material for: Effect of the At-CDC27a gene on Nicotiana benthamiana phenotype and accumulation of recombinant proteins
Source: Front Plant Sci. 2022 Nov 8;13:1042446. doi: 10.3389/fpls.2022.1042446 (PMC9679211; doi:10.3389/fpls.2022.1042446)
Supplement: Supplementary file 1 [file DataSheet_1.pdf]

Supplementary material

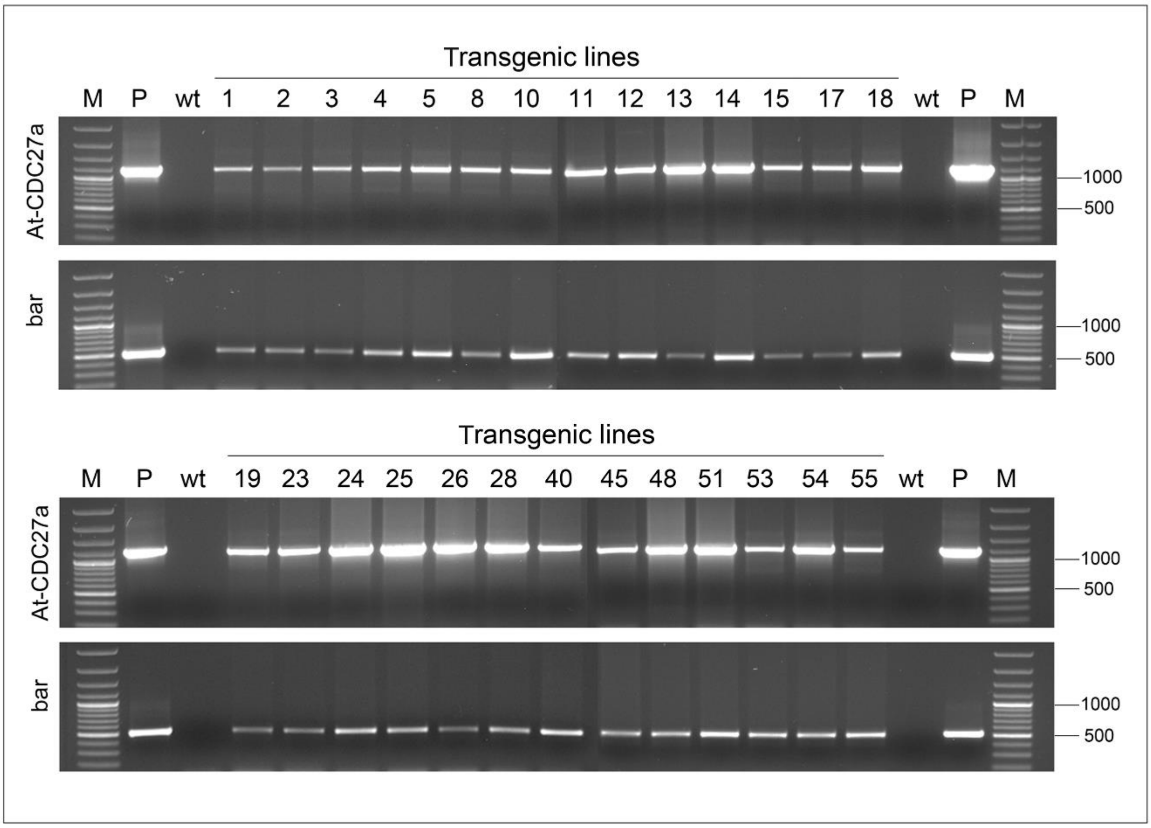

Figure S1. Verification of the At-CDC27a transgenic plants. (A) PCR analysis. Genomic DNA from the PPT resistant T_0 plants (1-5, 8, 10-15, 17-19, 23-26, 28, 40, 45, 48, 51, 53-54) was subjected to PCR analysis using the At-CDC27a-1200-forw/At-CDC27a-1200-rev and Bar-forw/Bar-rev primers specific to the *At-CDC27a* and *bar* genes, respectively. The *At-CDC27a* primers amplify a 1200 bp fragment of the corresponding gene, whereas the *bar* primers produce a 503 bp fragment. DNAs of non-transgenic *N. benthamiana* plant (wt) and plasmid pLH-*35S-At-CDC27a* (P) were included as negative and positive controls, respectively. GeneRuler 100 bp Plus DNA marker (Thermo Scientific, Waltman, United States (M).

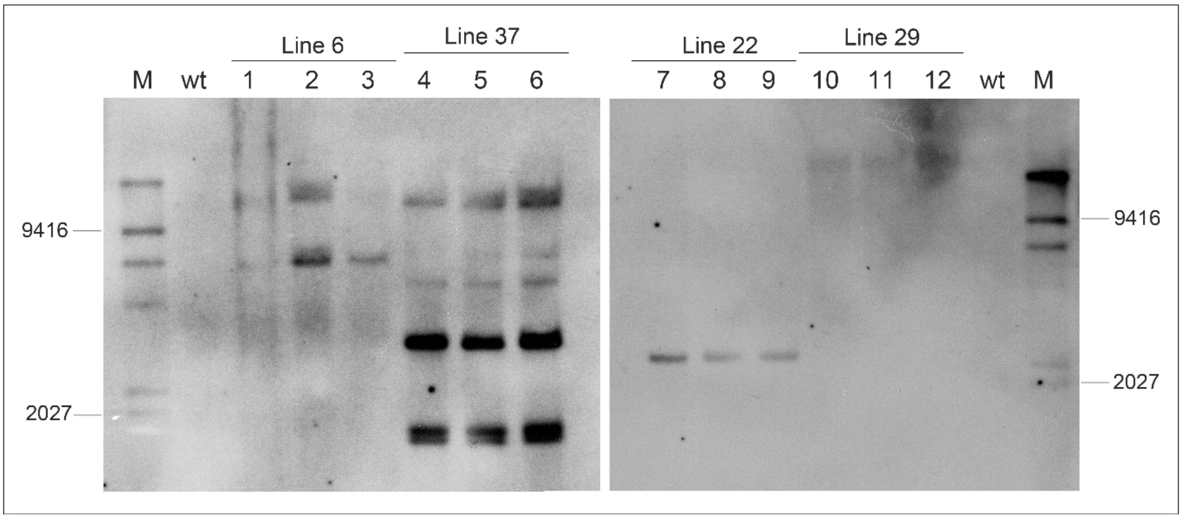

Figure S2. Verification of the At-CDC27a transgenic plants by Southern blot analysis.

Genomic DNA from non-transgenic and T1 progeny of the At-CDC27a transgenic plants (lines 6, 22, 29 and 37) was cleaved with *Bam*HI and hybridized with the DIG-labelled *bar* probe. Lanes are as follows: wild type *N. benthamiana* (wt), T1 transgenic plants (1-12), DIG-labelled molecular marker (M).

Tables

Table S1. Primers used in this study. Primers which were used for transgene verification (TV), expression analysis (EA) and development of probe for Southern blot analysis (SB) are presented.

Primer	Sequence (5'-3')	Application
Bar-forw	ATGCCGGCGGTCTGCACCATCGTC	TV, SB
Bar-rev	CGCGTGATCTCAGATCTCGGTGAC	TV, SB
At-CDC27a-1200-forw	ATGGAGAATCTACTGGCGAATTGTG	TV
At-CDC27a-1200-rev	GTACATATGTAAATGCCTGTGGCCA	TV
At-DC27a-511-forw	CGTAGTGCTAGAATAGCAGCAAGGA	EA
At-CDC27a-511-rev	CAGTGGAGTATGTATCCATTCCTTC	EA
GAPDH-238-forw	GGAGGAGGGAACAACAAGAGG	EA
GAPDH-238-rev	AGATGCCGTCAGTGCCGA	EA
GFP-forw	TACAAGACGCGTGCTGAAGT	EA
GFP-rev	CAATGTTGTGGCGAATTTTG	EA
TM43-E10-forw	GTTCTGGAAGCAGCTCCAAC	EA
TM43-E10-rev	ACCAGTCCATTCAGGCTGTC	EA
ubi-forw	CGACTCTTCATCTCGTGCTC	EA
ubi-rev	TGACGGTCAAAGTGGTTAGC	EA

Table S2. Transient production of GFP and scFv-TM43-E10 proteins in non-transgenic and At-CDC27a-29 plants. For RNA quantification 10 samples were evaluated for each variant (wt and At-CDC27a-29 plants). For protein quantification 6 and 8 samples for GFP and scFv-TM43-E10, respectively were collected at 4 dpi and analysed. Each sample is a pooled sample generated by combining three infiltrated spots from middle leaves of one plant. Values represent the means with SEM.

Genotype	GFP		scFv-TM43-E10	
	RNA, relative accumulation	Protein, µg/g	RNA, relative accumulation	Protein, µg/g
Wt	8560±753	196.2±14.3	1627±209	183.1±17.6
At-CDC27a-29	12578±624	422.7±39.5	2682±274	361.5±19.5

Table S3. The biomass and protein expression yields calculated for this study

Genotype	Leaf biomass fraction, g		GFP accumulation, mg/g		scFv-TM43-E10 accumulation, mg/g	
	1 plant	80 plants	1 plant	80 plants	1 plant	80 plants
Wt	9.4	752	1708	1284.4	0.2	150.4
At-CDC27a-29	13.0	1041.06	2491	2593.1	0.36	375.8

Methods

Southern blot analysis of the At-CDC27a transgenic lines

To determine the number of T-DNA inserts, genomic DNA was extracted from leaf tissue of transgenic and non-transformed plants by DNeasy Plant Maxi Kit (Qiagen, Hilden, Germany). Southern blot analysis was performed with 16 µg of DNA after restriction with *Bam*HI. The restricted DNA was separated by 0.8% agarose gel and UV coupled to a Hybond N+ nylon membrane. Blots were hybridized with a *bar* probe, labelled with a PCR DIG Probe Synthesis Kit (Merck, Darmstadt, Germany). Table S1 presents the *bar* primers used for generation of the DIG labelled *bar* probe. The membranes were developed and detected with DIG Detection system (Merck, Darmstadt, Germany) using conditions suggested by the supplier.
